# Statement of Retraction: Clinical practice of epidermal growth factor receptor-tyrosine kinase inhibitor targeted drugs combined with gadolinium oxide nanoparticles in the treatment of non-small cell lung cancer

**DOI:** 10.1080/21655979.2024.2299626

**Published:** 2024-02-20

We, the Editors and Publisher of the journal *Bioengineered*, have retracted the following article:

Xuan Zhou, Ting Jin, Likun Wang, Erlin Zhao & Xuyang Xiao (2022) Clinical practice of epidermal growth factor receptor-tyrosine kinase inhibitor targeted drugs combined with gadolinium oxide nanoparticles in the treatment of non-small cell lung cancer, Bioengineered, 13:1, 128-139, DOI: 10.1080/21655979.2021.2009969

Since publication, significant concerns have been raised about the integrity of the data and reported results in the article. When approached for an explanation, the authors did not provide their original data or any necessary supporting information. As verifying the validity of published work is core to the integrity of the scholarly record, we are therefore retracting the article. The corresponding author listed in this publication has been informed.

We have been informed in our decision-making by our editorial policies and the COPE guidelines.

The retracted article will remain online to maintain the scholarly record, but it will be digitally watermarked on each page as ‘Retracted’.

